# High-power motorcycle accidents in Spain: a descriptive study

**DOI:** 10.1007/s00068-023-02363-0

**Published:** 2023-09-12

**Authors:** José Miguel Díez-Navarro, Cesar Leal-Costa, David Planes-Muñoz, María Suárez-Cortés, María de los Ángeles Castaño-Molina, Alonso Molina-Rodríguez, José Luis Díaz-Agea

**Affiliations:** 1 Health Sciences PhD Program, Universidad Católica de Murcia UCAM, Campus de los Jerónimos n_135, Guadalupe, 30107 Murcia, Spain; 2 Edificio LAIB/DEPARTAMENTAL, Faculty of Nursing, University of Murcia, Campus de Ciencias de la Salud, El Palmar-Murcia, 30120 Murcia, Spain; 3 Food Science and Human Nutrition Department, Faculty of Veterinary, Regional Campus of International Excellence “Campus Mare Nostrum”, University of Murcia, Campus de Espinardo, 30100 Murcia, Spain

**Keywords:** Motorcycle accidents, Mortality, COVID-19, Medical assistance, Socioeconomic factors

## Abstract

**Purpose:**

In modern societies, motorcycle accidents have become a great problem for health systems worldwide. In Spain, the size and the power of the engine of 2-wheel vehicles determine the type of driving license and the age at which these vehicles can be used (mopeds and motorcycles, which at the same time can have a small or large engine capacity). The objective of the present study was to analyze and characterize low- and high-power motorcycle accidents in Spain, between 2014 and 2020, both included and compared these categories with each other.

**Methods:**

Retrospective, descriptive, and observational study of motorcycle and moped accidents in Spain between 2014 and 2020, both included.

**Results:**

The mortality of motorcycle accident riders in Spain is associated with males aged between 30 and 40 years old, with a high-power motorcycle, and an A or A1 driver’s license, who is 6.7 times more likely to die in crossings and highways than a moped, while wearing a helmet, and if not, this increases to 4.89 times. During the COVID-19 pandemic, an increase in death at 24 h after a high-power motorcycle accident was observed, as compared with a large reduction in the total medical assistance provided in 2019–2020.

**Conclusions:**

High-power motorcycles had higher scores in mortality and morbity rates than low-power ones, with a significant increase in mortality during the pandemic, even though number of accidents and medical assistance provided were drastically reduced.

## Introduction

In the last few decades, due to the increasing number of motorcycles on roads, it is evident that traffic accidents in which a motorcycle is involved, it has become one of the greatest health problems in modern societies.

Worldwide, the recent changes in the type of economy, the increase in the price of fossil fuels, the modernization of societies, the difficulty in finding parking in large urban centers, and the established concept of an “eco” way of life, have defined motorcycles as less contaminating means of transport, unseating passenger cars as a day-to-day use vehicle, especially mopeds and low-power motorcycles.

Statistical studies [1, 2] have shown that this is not a trivial problem, as motorcycle accidents are the 8th leading cause of mortality worldwide, and the first if we focus on the 15–29 age range. At the worldwide level, the figures of the deceased are around 1.2 million individuals, and more than 50 million injured due to diverse causes associated with these accidents.

The World Health Organization (WHO) points out a disparity when comparing these figures in underdeveloped societies, as they are much higher there. If urgent plans and policies are not implemented, motorcycle accidents could even become the 5th leading cause of death due to traffic accidents by 2030.

Concerning motorcycle and moped users, it can be inferred, according to [3], the number of motorcycles has considerably increased, by 41% in the European Union. An Australian study [4] confirmed that between 2007 and 2012, the number of registered motorcycles increased by 33%, with this increase being 3 times higher when compared to automobile users.

Contrary to all the advantages described above, motorcycle users are also very vulnerable. Statistical analyses in Europe have shown that the risk of being injured or dying, due to riding a motorcycle, when traveling the same distance as a passenger car, is 18 times higher [1]. Other articles [3] indicate an increase of 15% in fatal motorcycle accidents.

In France, motorcycles comprise 1.5% of all the traffic on highways, but if we focus on more cosmopolitan areas, with other road users, the percentage is higher. The number of motorcycle users in large towns has increased abruptly, especially in areas near Paris and its surroundings. More specifically, it is estimated that in this decade, the city of Paris saw an increase of 64% in the number of motorcycles [5].

The data from the United States show an increasing number of motorcycles as well, from 4.26 million in 1990 to 6.69 million in 2006, with motorcycle sales also increasing, from 278,000 in 1992 to 1.1 million in 2007 [6].

In 1997, the Australian Federal Office of Road Safety published that the risk of suffering severe injuries, or even fatal ones when riding a motorcycle, was around 20 times higher than in passenger cars, for the same distance traveled, as compared to car drivers [7, 8].

North American studies, from the National Highway Traffic Safety Administration, in 2007, estimated that the probability of dying from a motorcycle accident, when compared to a car, by mile traveled, was 34 times higher. Also, they indicated that the risk of suffering from lesions was 8 times higher as compared to other types of motor vehicles [9].

As for Spain, an increase has been found in the last few years, in agreement with the rest of the countries. The numbers have increased from 1,445,644 registered motorcycles in the year 2000, to a total of 2,891,204 in 2013, according to the General Directorate of Traffic (Dirección General de Tráfico, DGT). This increase has been influenced by the entry into force of Royal Decree 1598/2004, July 2nd, which, following the text found in the 91/439/CEE Directive, modifies the General Guidelines for Drivers, allowing drivers with a B license for more than 3 years, to ride motorcycles allowed by the A1 driver’s license [10].

After the review of the studies consulted, the current situation of morbi-mortality associated with the use of motorcycles is evident, for both leisure and utilitarian use. There are risk factors and prevalent injuries inherent to the collective. A descriptive study on accidents, as well as the possible correlations of these factors, could be a great tool that could be used by institutions associated with the prevention and protection of this sector of society.

The common imagination has pigeonholed high-power motorcycle users as being adult male, with a medium purchasing power, who rides motorcycles irresponsibly, increasing the risk of having an accident, resulting in death, or injuring others. Does this belief have an objective basis? In the present article, we will try to provide an answer to this question, by analyzing the profile of motorcycle accidents within a range of 7 years.

## Materials and methods

### Objectives

#### General objective

To analyze and characterize low-power and high-power motorcycle accidents in Spain between 2014 and 2020, both years included, and to compare these categories with each other.

#### Specific objectives

1. To determine and compare the sociodemographic characteristics of low and high-power motorcycle riders in Spain between 2014 and 2020.
2. To determine and compare the more prevalent risk factors that intervened in low and high-power motorcycle accidents in Spain between 2014 and 2020.
3. To compare the severity of the low and high-power motorcycle accidents in Spain between 2014 and 2020, both years included, in relation to the variables vehicle, rider, and most prevalent accidents.
4. To analyze and compare the evolution of low- and high-power motorcycle accidents.

### Population

This study is based on data obtained from the General Directorate of Traffic in Spain, in the 2014–2020 period, from the entire Spanish territory, sent by the Statistical Service from the National Observatory of Road Safety. We requested the database from the Spanish Directorate General of Traffic. Our request was submitted to the General Directorate of Traffic (DGT) on January 9th, 2022, through the Transparency Portal, under the provisions of Law 19/2013, dated December 9th, which pertains to transparency, access to public information, and good governance. The registration number for our request is 001-064346.

These data were collected by the agents in charge of traffic control and management during the accident. The present work analyzed 7 traffic accident databases provided by the Spanish DGT. These 7 reports were merged into a database which ultimately contained 173,729 entries. For this work, the data was filtered by type of vehicle, leaving only motorcycles < 50 cc (low power), and > 125 cc (high power), with the resulting database having a total of 115,135 entries corresponding to accidents.

### Research design

A cross-sectional, retrospective, exploratory, and descriptive study of motorcycle and moped accidents that occurred in Spain during the 2014–2020 period, both years included [11].

### Source of data

In 2014, Order INT/2223/2014, from October 27th, was updated, which regulates the communication of information to the National Registry of Traffic Accident Victims.

The questionnaire, completed by the security forces who helped after the accident, was divided into three sections:

1. General data on the accident, such as date, time, type of accident, location, etc.
2. Data on the vehicles involved, such as the type of vehicle, number of occupants, state of the vehicle, etc.
3. Data on the people involved, such as actions by the rider, violations, injuries, etc.

The questionnaire was reviewed to avoid omissions or mistakes during its completion; it was sent, within five days after the accident, to the designated Provincial Traffic Headquarters, according to the location of the accident. When it was sent, it was previously verified if there were injured individuals, and their state in the first twenty hours of the accident, to discover if a death had occurred within the first few hours after the accident, or if there were any minor or severe injuries. This is when health institutions become relevant, as they are responsible for providing data related to injuries or the state of the injured.

Once the health data were obtained, the report was copied and sent to competent bodies from the Spanish Government Ministry of Public Works and Transports. Then, within the first fifteen days of the accident, the data were entered into the Central Services of the General Directorate of Traffic.

### Data analysis

The seven databases of traffic accidents in Spain, corresponding to the years between 2014 and 2020, were merged into a database, after which it was filtered according to the two types of vehicles of interest for the present work, lower-power motorcycles (< 50 cc), and high-power motorcycles (> 125 cc). The descriptive statistics are shown as mean ± standard deviation for the quantitative variables. The analysis of the accidents between the low and high-power motorcycles, and other parameters of interest, was performed through logistic regression models, to obtain results, such as odds ratio (OR) and confidence interval (CI) at 95%. The death rates were calculated as the number of deaths per 100 victims. All the analyses and figures were created with the R version 4.2.2 statistical software, with *p* value < 0.05 utilized to determine statistical significance.

## Results

Seven databases were utilized, each corresponding to a different year (2014–2020), with information relative to the riders, and a single database was created with 115,135 entries, after debugging the database. This database was used to analyze the information of the riders, as a function of the type of motorcycle used during the accident.

### Statistical description of the riders

#### Age of the riders according to sex and type of motorcycle

In the low-power category, the mean age of the men was 36.79 years old, with a standard deviation ± 16.82 years. The women had a mean age of 35.71 years old, with a standard deviation ± 13.55 years. The overall mean age (men and women) was 36.51 years old, with a standard deviation ± 15.99 years. Table 1 shows the mean ages of the riders who suffered accidents, for both lower-power and high-power motorcycles.

**Table 1:** Mean age according to sex and type of motorcycle of drivers who suffered accidents, from 2014 to 2020

Sex	Low power	High power
Men	36.79 ± 16.81	41.72 ± 13.81
Women	35.71 ± 13.35	38.88 ± 12.34
Total	36.51 ± 15.99	41.24 ± 13.61

#### Distribution according to age and frequency according to low or high-power motorcycles

**Fig. 1:**
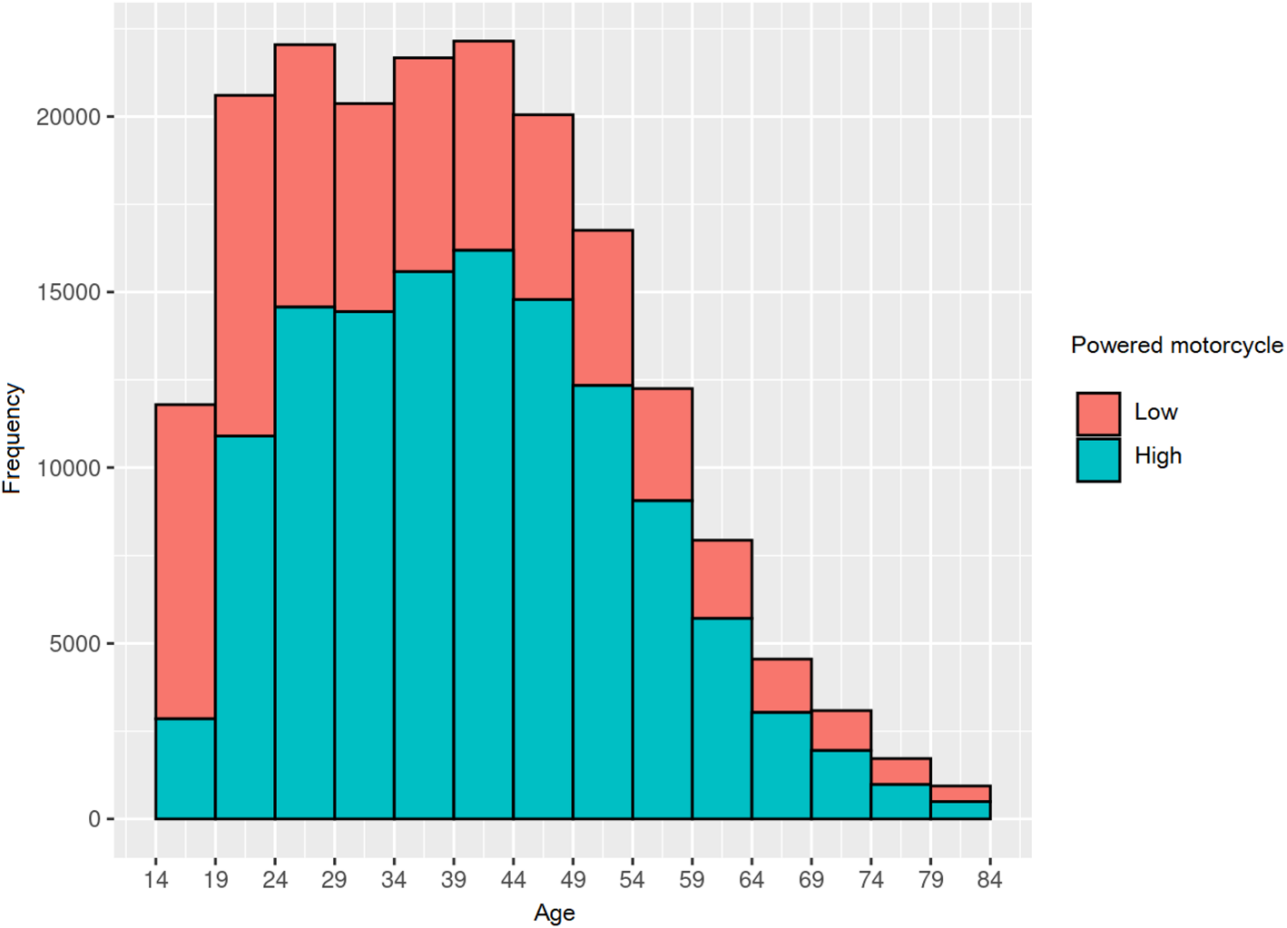
Distribution of age and frequency according to low or high power

The age of the drivers who suffered an accident is shown grouped according to the type of motorcycle, age, and sex. The data are presented as mean ± standard deviation. Also, a histogram of the frequencies of the ages during the accident is shown, grouped according to the type of motorcycle (Fig. 1).

#### Use of helmet according to area and power of a motorcycle.

**Fig. 2:**
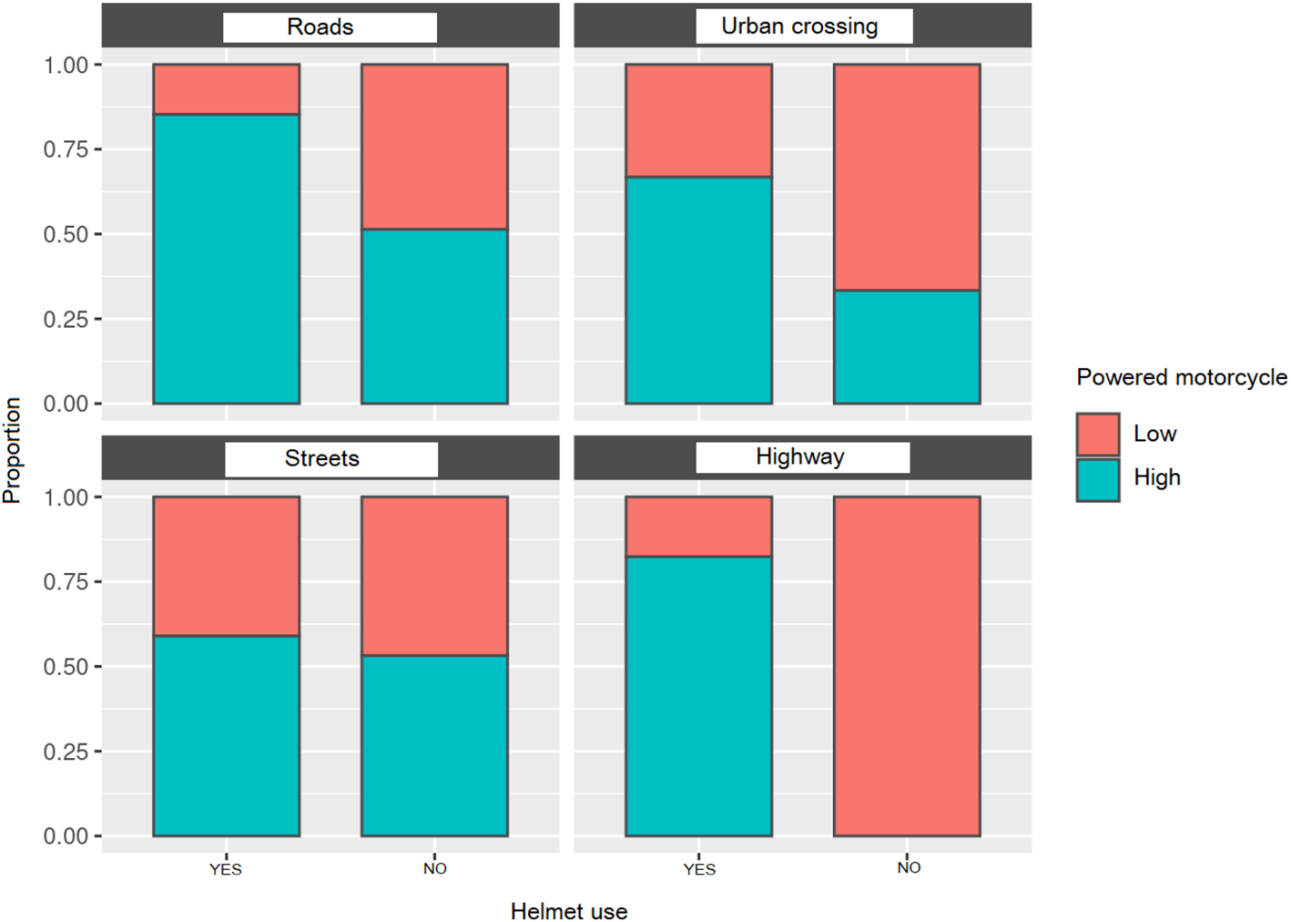
Helmet use (yes/no) as a function of the power of the motorcycle, expressed as a proportion

In the following figure, helmet use was analyzed depending on the power of the motorcycle and the different types of roads. The data are expressed as proportions. There was no data for the category low power and highways, as in Spain, it is illegal for these vehicles to use them (Fig. 2).

#### Helmet use as a function of the type of driver’s license and motorcycle power

**Fig. 3:**
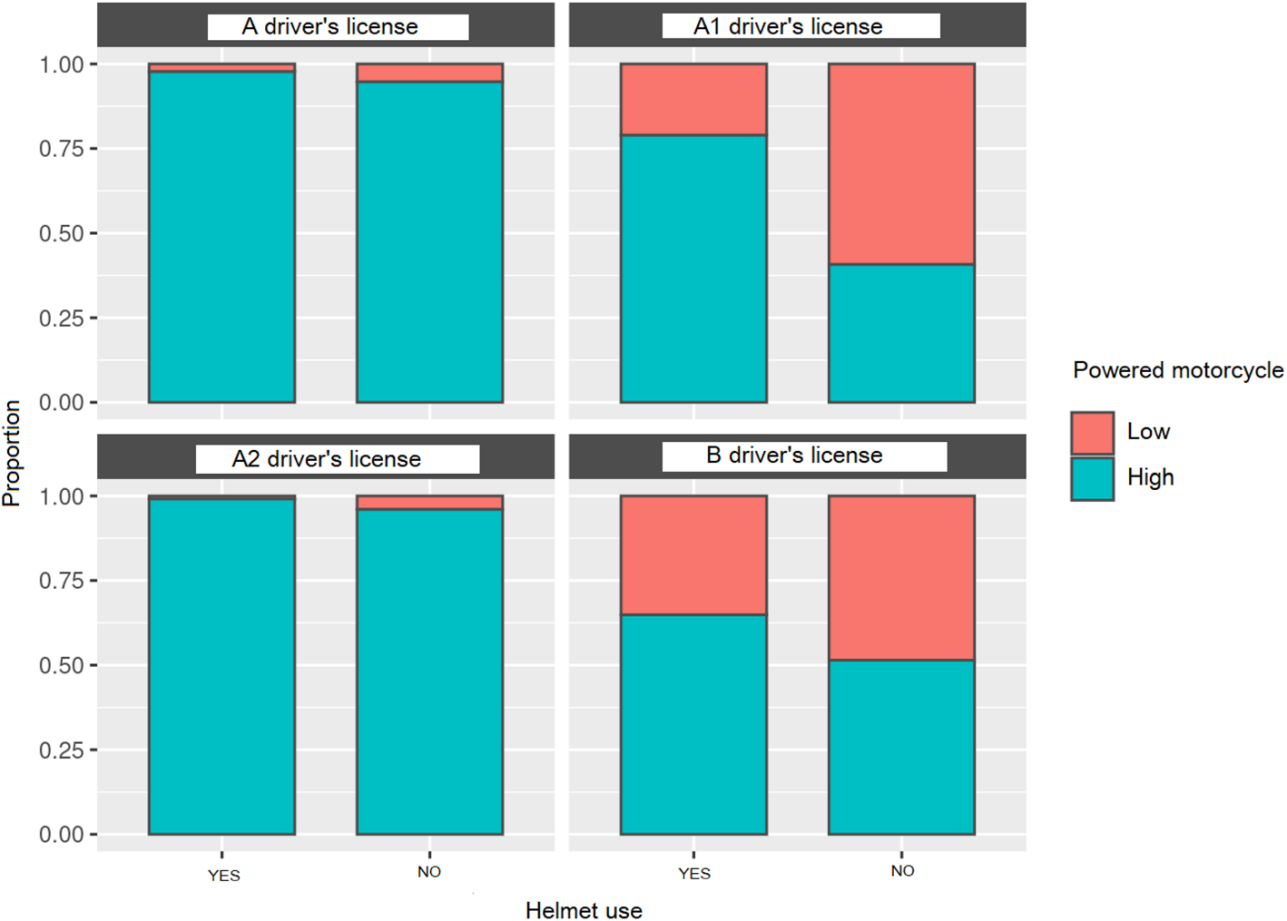
Helmet use as a function of the type of driver’s license and motorcycle power expressed as a proportion

The figure below visually shows helmet use according to the type of valid driver’s license during the accident and the type of motorcycle. As defined by the Dirección General de Tráfico, in Spain, the different types of licenses are class “A”, which allows driving any type of motorcycle and motor tricycle. To obtain the “A” license it is a requirement to be at least 20 years old and to have held the “A2” license for at least two years. The “A2” license allows driving motorcycles with a maximum power of 35 kW, and a minimum age of 18 years. The “A1” license allows driving motorcycles with a maximum power of 11 kW, with a minimum age of 16 years. Car drivers, who have a class “B” license and have had it for 3 years, may drive motorcycles of category “A1”. It can be observed that there was a broad use of helmets by riders with an “A” driver’s license. For the type “A2” license, the same correct use concerning the “A” category was observed. In the “A1” license, a clear decrease in the use of helmets was observed in low-power motorcycles and a slight decrease in high-power ones. Lastly, for the riders with the “B” license, a clear decrease was observed in the use of helmets in both low-power and high-power categories (Fig. 3).

### Analysis of mortality of riders

#### Mortality rate according to sex

Table 2 below shows the accident rates according to the sex of the drivers and their membership to the low-power and high-power categories (see Table 2).

**Table 2:** Death rate according to sex

Ratios	OR	95% CI		*p*
Women (high/low)	1.252	1.223	1.281	0.000
Men (high/low)	2.194	2.167	2.222	0.000
Odds ratio (men/women)	1.753	1.707	1.800	0.000

The women obtained an accident rate of 1.252 higher in high-power motorcycles as compared to low-power ones (OR 1.252; 95% CI 1.22–1.28; *p* < 0.001). Meanwhile, men had an accident rate 2.194 times higher with high-power motorcycles than with low-power ones OR 2.194; 95% CI 2.17–2.22; *p* < 0.001). When the variables men and women were compared, we obtained an accident rate 1.753 times higher for men with respect to women, which indicates that men had a 75.3% higher probability of being involved in an accident as compared to women, with high-power motorcycles (OR 1.753; 95% CI 1.71–1.80; *p* < 0.001).

#### Death of riders within 24 h

The table below shows the proportions of death within the first 24 h (see Table 3).

**Table 3:** Proportions of the deceased in the first 24 h

Proportions	OR	95% CI		*p*
No (high/low)	1.949	1.923	1.975	0.000
Yes (high/low)	6.714	5.529	8.154	0.000
Odds ratio (yes/no)	3.446	2.836	4.186	0.000

The probability of not dying within the first 24 h after an accident while riding a high-power motorcycle concerning a low-power one was 1.949 times more (OR 1.949; 95% CI 1.923–1.975; *p* < 0.001). The probability of dying within the first 24 h from an accident while riding a high-power motorcycle concerning a low-power one was 6.714 times more (OR 6.714; 95% IC 5.529–8.154; *p* < 0.001). When the variables were adjusted, the probability of dying within the first 24 h from the accident while riding a high-power motorcycle was 3.5 times higher (OR 3.446; 95% CI 2.836–4.186; *p* < 0.001).

**Fig. 4:**
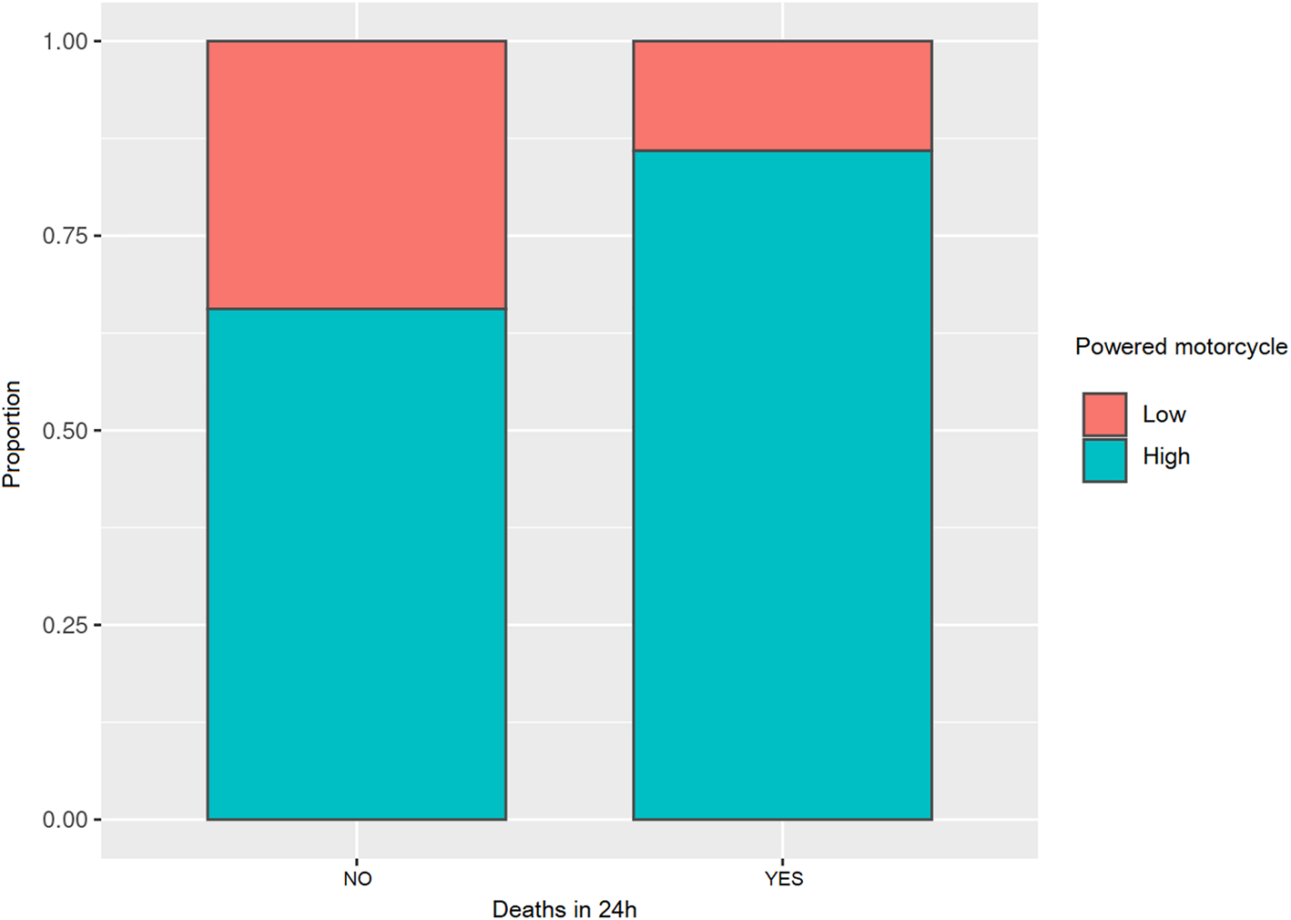
Deaths in the first 24 h according to riders and motorcycle power as a proportion

Figure 4 more clearly shows these data as a proportion.

#### Index of lethality in 24 h according to sex, nationality, helmet use, and type of license

Next, the lethality indices were analyzed 24 h after the accident, focusing on if they belonged to the low-power or high-power categories (Table 4).

**Table 4:** Index of lethality in 24 h according to sex, nationality, helmet use, and type of license

	Low-power	High-power
Men	0.44	1.37
Women	0.07	0.19
Spanish	0.43	1.2
Foreign	0.27	1.52
Helmet no	2.84	4.89
Helmet yes	0.46	1.95
License A1	0.14	1.3
License A	0.31	3
License B	0.25	0.76
License A2	0	2.45

#### Index of lethality in 24 h according to the area of the accident

The following table shows the result of the lethality index in 24 h according to the type of motorcycle (low or high power), and if the accident took place in a road, crossing, street, or highway. For the category low power and highways, this value is “0”, as mopeds are not allowed to use them (Table 5).

**Table 5:** Lethality indices in 24 h according to the area, of drivers

Area	Low-power	High-power
Road	1.62	2.62
Urban crossing	0.51	1.66
Street	0.18	0.43
Highway	0	4.76

### Analysis of the increase in mortality in high-power motorcycles coinciding with the COVID-19 pandemic

In the following section, an in-depth analysis is made of the data on the significant increase in mortality of high-power motorcycle riders who suffered an accident during the pandemic in Spain.

**Fig. 5:**
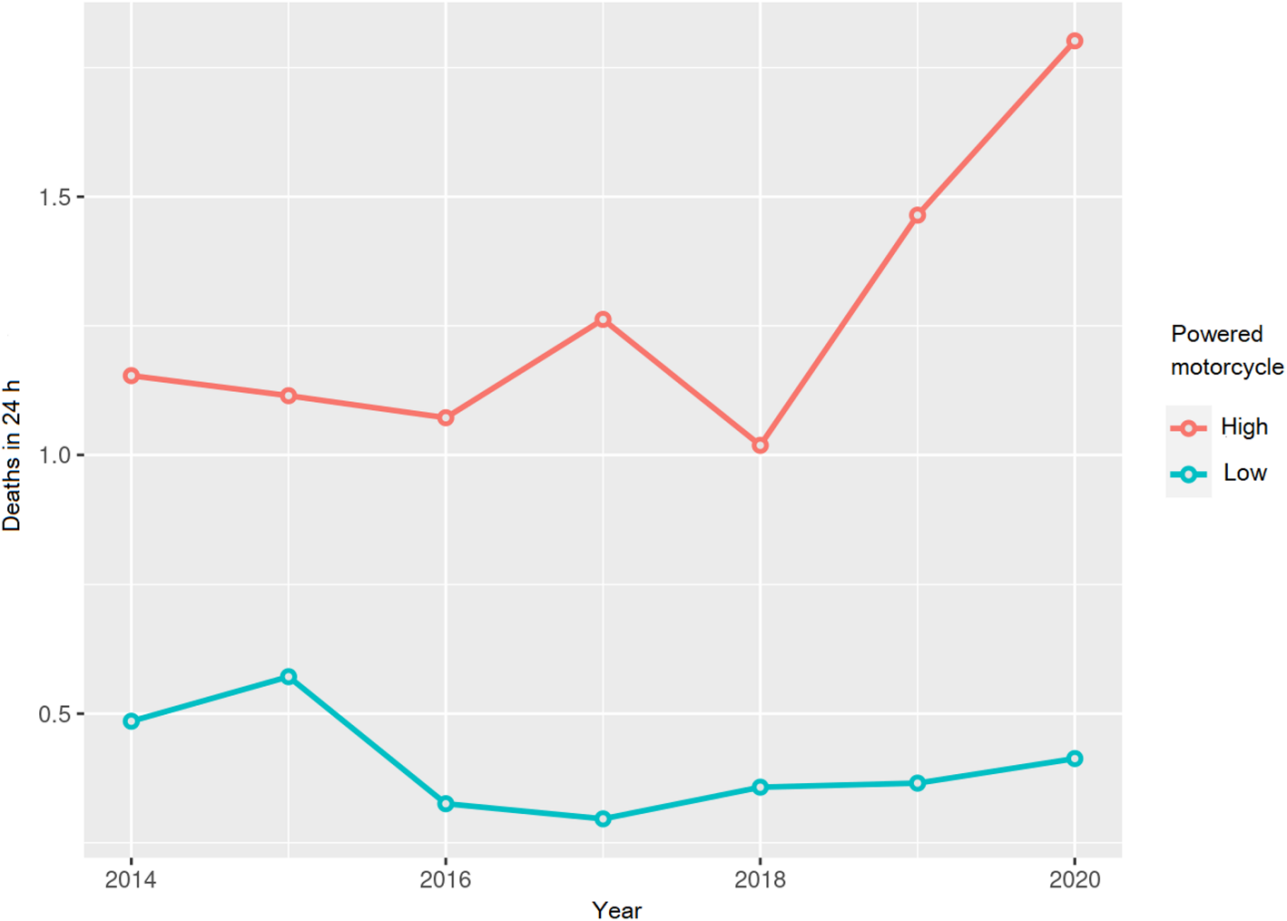
The ratio of deaths in the first 24 h, according to year and power, is expressed as a proportion

The same data are shown in Fig. 5 as a proportion, for death in 24 h and year.

**Fig. 6:**
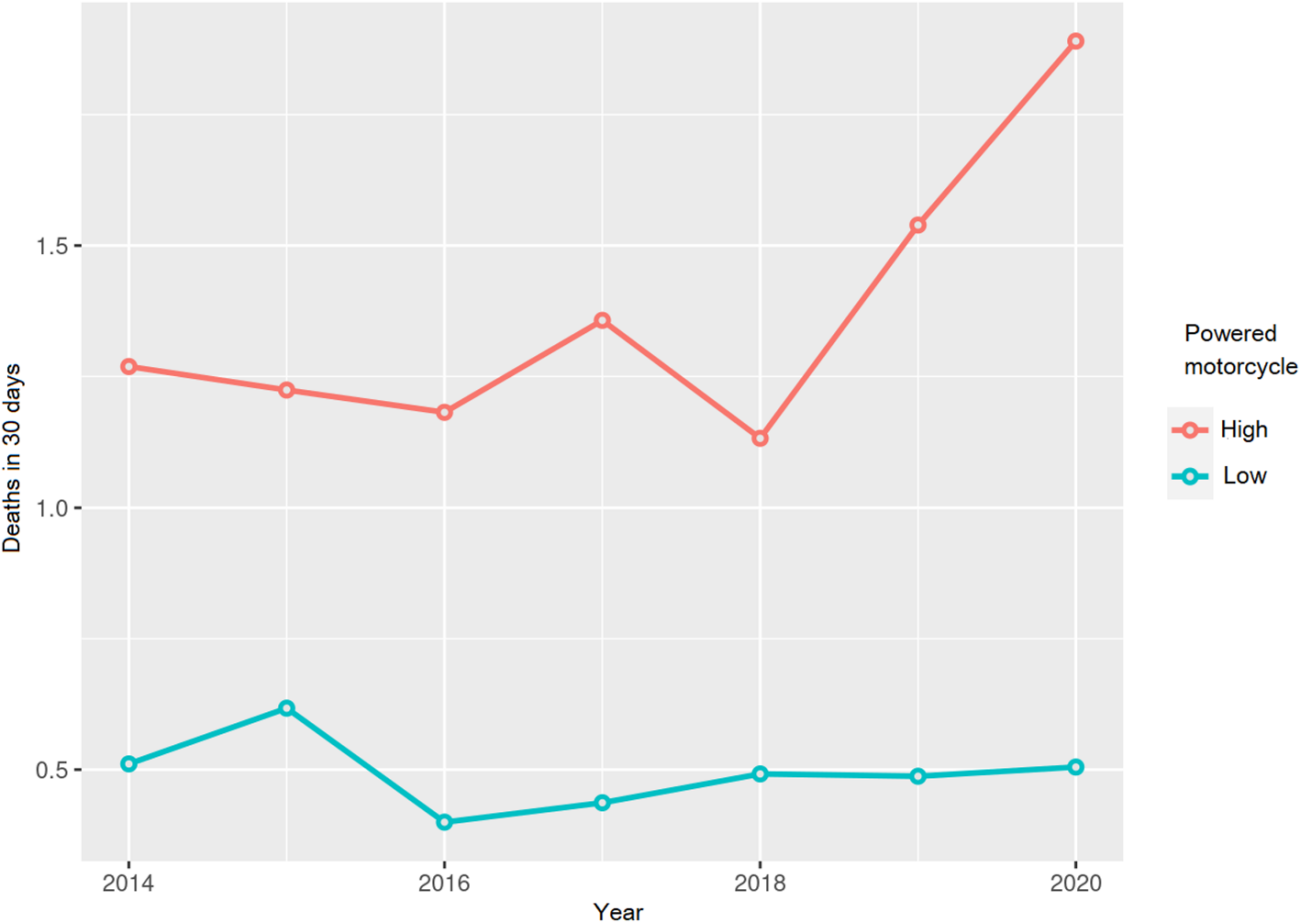
The ratio of deaths in the first 30 days after the accident and year, is expressed as a proportion

The same data are shown in Fig. 6, as a proportion, for deaths in the first 30 days after the accident and year, with an increase observed in the number of deaths in accidents involving a high-power motorcycle.

Table 6 shows the number of deceased, who died in the first 24 h, and the total medical care provided per year.

**Table 6:** Total number of accidents, deaths in 24 h, and medical care provided from the total motorcycle riders who suffered an accident, per year

Year	Accidents	Deaths in 24 h	Care provided
2014	6894	86	4478
2015	7933	103	5007
2016	11,239	113	7562
2017	10,902	140	6901
2018	11,987	126	7546
2019	8855	130	5590
2020	6599	110	4279

**Fig. 7:**
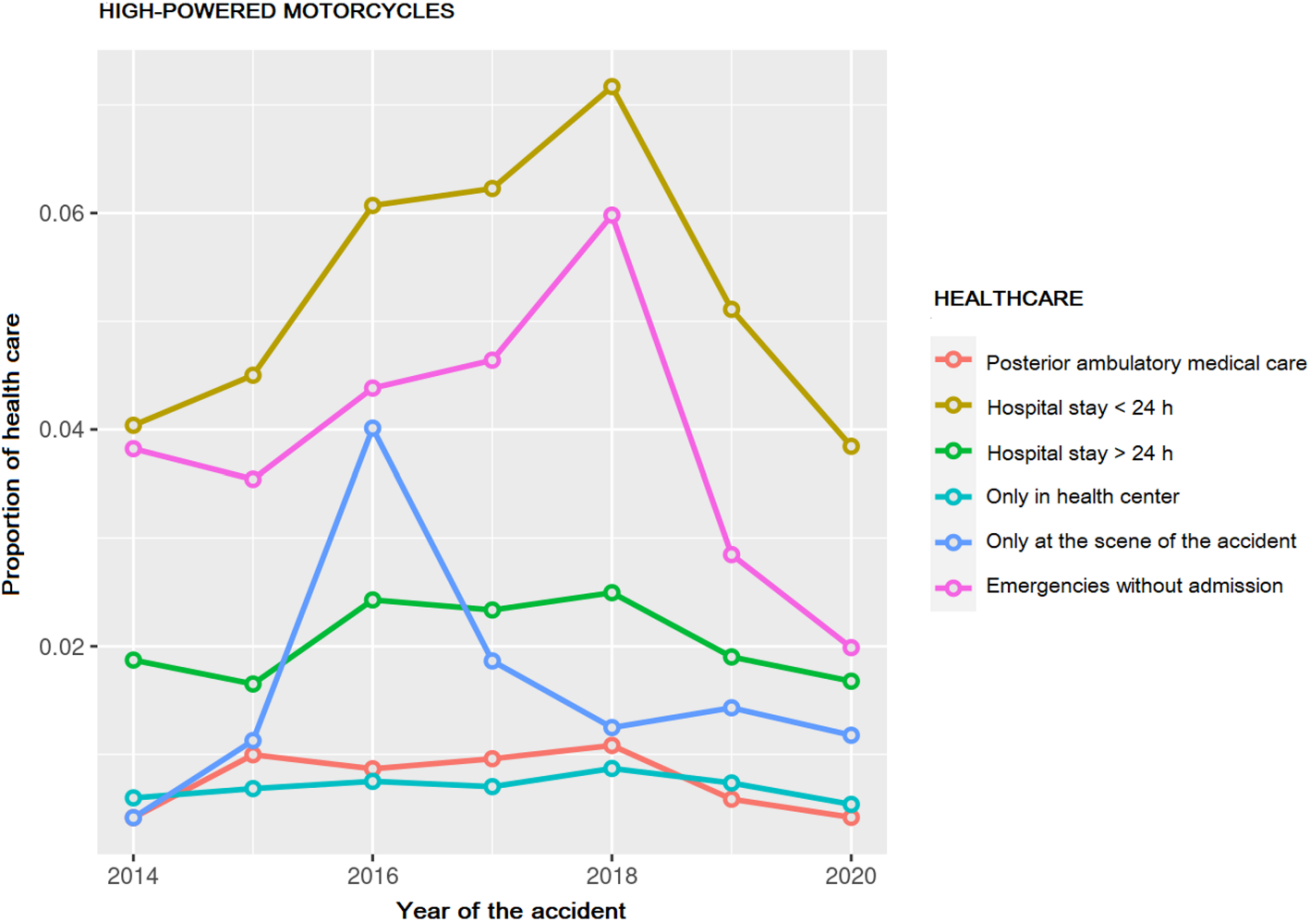
Type of more critical care, in high-powered motorcycles, per year and expressed as a proportion

Posteriorly, the type of assistance given to high-power motorcyclists was analyzed. The results are shown in figures, as they are more intuitive to understand, with the data expressed as a proportion (Fig. 7).

## Discussion

Based on the data from the DGT analyzed, a pattern was observed of low-power and high-power motorcycle users, for accidents between the years 2014 and 2020, both included, in Spain. Likewise, the main risk factors present during the accident were found, as well as their significant influence on its occurrence.

The mean age of the high-power motorcycle rider was 41.24 years old, while the mean age of the low-power motorcycle was 36.51 years old, which at first does not match with the scientific literature available, which indicates that younger and older individuals had a higher propensity for having a motorcycle accident [12–14]. In the specific case of high-power motorcycle riders, being younger was an important and specific risk for having accidents [15–18].

The analysis of the distribution of frequencies based on age when using low- or high-power motorcycles showed a clear use of low-power motorcycles by the age group comprised of 14- to 19-year-olds. In the 19–24 age range, the preference for the use of low- or high-power motorcycles was the same. And in the case of the 24–74 years old age range, the use of high-power motorcycles strongly increased. In the older age group, a clear decrease was observed in the use of high-power motorcycles, down to values that were similar between low and high-power motorcycles.

According to the sex of the riders, most were men (79.4%), in agreement with other studies [1, 19]. The probability of having an accident, being male, and with a high-power motorcycle, compared to being a woman, was 2.194 higher. This agrees with a Spanish study on moped accidents [1]. The probability of having an accident with a high-power motorcycle was greater for men as compared to a low-power motorcycle, 1.37 times as compared to 0.44 with a low-power one. The probability of accidents with a high-power motorcycle was 1.754 times higher for men when comparing it to women. Being a woman obtained better overall results with respect to accidents.

There were practically no differences in accidents in the use of low or high-power motorcycles, with a death rate of only 0.19 times higher for high-power motorcycles, in agreement with other literature consulted [20, 21]. However, other studies were not in agreement, as they indicated that women were more likely to have accidents, with more severe injuries, and higher death rates [21, 22].

Table 4 and Fig. 5 show the rates and proportions of death at 24 h. The results for the high-power motorcycles as compared to low-power ones are striking, as the probability of dying from an accident with a high-power motorcycle is 6.7 times higher. The odds ratio for the probability of dying or not, in the first 24 h after an accident with a high-power motorcycle, was 3.446 times higher.

The use of helmets depending on the type of road results showed that the low-power motorcycle riders tended to not use helmets in crossings and roads. The high-power motorcycle riders tended to use helmets when circulating on fast roads, such as urban crossings, freeways, and highways. The probability of dying due to not wearing a helmet during the accident was 4.89 times higher than when using it. The low-power motorcycles did not obtain a score for highways, as motorcycles smaller than 125 cc are not allowed to circulate in them. When analyzing helmet use according to the type of license, it was observed that those with a “B” driver’s license (both types), for both low and high-power motorcycles, used helmets the least, while those with driving licenses specific for motorcycles, “A” and “A2”, obtained the best helmet use results. This indicates that those who had a motorcycle-specific license had a better understanding of the risk of their use and the possible injuries they could suffer from. Thus, the implementation of the use of high-power motorcycles without specific licenses must be carefully studied due to the risk it could imply for the safety of the users.

Although the highest number of accidents occurred in urban areas, it must be underlined that the data obtained in the present study showed that the injuries were less severe, and the probability of dying in the first 24 h of the accident in urban areas was lower. These rates strongly increase when focusing on interurban areas. The probability doubles when riding a high-power motorcycle as compared to a low-powered one. And it quadruples when comparing high-power motorcycles and low-powered ones on highways, up to 4.76 times. This agrees with other studies consulted, both European [23–26], as well as from other continents [27, 28]. Several studies have corroborated that implementing additional lanes, augmenting lane width, or enhancing pavement conditions may yield advantageous outcomes in terms of safety [29, 30]. The high index of injuries obtained on highways leads us to think about the possibility that these pavement suitability factors could incite motorcycle riders to increase their level of risk and aggressiveness when riding, as pointed out in other studies [31, 32].

When analyzing the type of license, contradicting results were observed. Just as the discussion above on the use of helmets, as the scale of the licenses increased, from B to the highest A, the use of helmets also increased, perhaps indicating that the rider’s awareness of passive protection increased. When analyzing the death rates after 24 h, worse data were observed for A and A1 licenses, with death rates 3 and 2.45 higher in high-power motorcycles, for 0.31 and 0 rates of these licenses and low-power motorcycles.

To conclude, a striking result from the study will be discussed: the increased death rates in 24 h during the coronavirus (SARS-CoV-2) pandemic.

As shown in Fig. 5, the death rates in 24 h for high-power motorcycle accidents were about 1.3 times between 2014 and 2018. Posteriorly, a significant increase was observed in the death rate at 24 h with high-power motorcycles, with the proportions increasing 2.0 times higher in the years that coincided with the pandemic (2019–2020).

In the categories of low-power motorcycles, this increasing trend in the death rate at 24 h was not observed, even decreasing or staying the same. These data could be considered normal, as during the pandemic, the exposure of motorcycle riders to accidents should have been less due to the confinement and the reduction in nonessential travel that took place in society.

In our study, it was found that during the years closest to the pandemic (2016–2018), the number of medical assistance provided to low- and high-power motorcycles was around 7300, with a mean of 126 deaths in 24 h. When focusing on the pandemic (2019–2020), as expected, given the reduction in social interactions, the mandatory confinement of society, and the decrease in on-essential travel, the mean of medical assistance to motorcycle riders who had an accident decreased to around 4900. When comparing these years with those described previously, there were 2400 fewer medical assistance events during the pandemic. In addition, when analyzing the number of deaths in 24 h during the pandemic, a mean of about 120 deaths in 24 h was observed.

As we can observe, the number of deceased during the COVID-19 period was similar to those before the pandemic, although the medical care provided to motorcycle riders who had had an accident decreased, due to the social health conditions imposed by the government. We asked ourselves why the number of deaths in 24 h was not reduced, considering the drastic decrease in medical assistance, as there was no evidence that the risk factors and types of accidents had substantially changed enough to influence mortality at 24 h. Given that the accidents had the same pattern as the previous years, and the physical medium in which they occurred had not improved either, made us hypothesize if the medical assistance could have had an influence, in that having 2400 fewer medical assistance events during the pandemic had not resulted in a decrease in mortality at 24 h, as compared to the pre-pandemic period (126–120).

The percentage of medical assistance events was analyzed separately for low-power motorcycles, without a significant effect found, so we focused on analyzing high-power motorcycles with an admittance longer than 24 h, admittance lower than or up to 24 h, and emergency care without a posterior admittance. A reduction in all types of medical assistance events in the database, as the overall data already indicated.

If we hypothesized that medical assistance during the pandemic influenced the non-reduction of mortality in 24 h, with less medical assistance provided, we need data on intensive care (intensive care unit, ICU) that had provided medical assistance to these patients. It must be remembered that in 2019 and 2020, healthcare infrastructures, and especially Intensive Care Units (ICU) were subjected to great medical care pressures, with many of them having to discriminate what patients could be admitted, with saturated floors and hospital emergencies overwhelmed. We contacted the Statistics Services of the DGT, to discuss this special case, although they responded that they did not have this type of data. This, our final analysis could not be conducted, although a new line of research was created to continue with this study and validate our hypothesis.

## Conclusions

In both high-power motorcycle accidents, as well as low-power motorcycle ones, the riders were predominantly male, between the ages of 30 and 40 years old. In the case of high-power motorcycles, there is even a sharper decrease in their use by women.

The probability of dying from using a high-power motorcycle and being a male was 2.2 higher than due to the use of a low-power motorcycle. When comparing men and women and the use of high-power motorcycles, the death rate was 1.753 higher for men.

According to the age group, a clear use of low-power motorcycles was found for ages between 14 and 19 years old. Posteriorly, the use of high-power motorcycles prevailed until the age of 74. As for those older than 74, the use of low- and high-power motorcycles was equal.

The use of helmets when using high-power motorcycles was double that of low-power motorcycles.

The use of helmets was lower in low-power motorcycles, and with individuals who did not have motorcycle-specific licenses (class B). Not using a helmet when riding a high-power motorcycle doubled the probability of dying in the first 24 h as compared to not using it when riding a low-power motorcycle. There was a propensity to not use a helmet when riding a low-power motorcycle through roads or urban crossings, as well as during leisure or entertainment use.

Most of the accidents that occurred in urban areas had a lower morbi-mortality rate than high-power motorcycle accidents on highways and freeways. The death rate was 4 times higher on these types of roads when using a high-power motorcycle.

There was a greater number of low-power motorcycle users with a type B license. The high-power motorcycles were associated with specific licenses.

Contrary to helmet use, we found increases in the rates of injuries with high-power motorcycles and type A and A1 licenses, with rates 3 and 2.45 times higher, respectively.

The death rates within 24 h, for high-power motorcycles, were around 1.3 times higher between 2014 and 2018. Afterward, a significant increase was observed in the death rate at 24 h for high-power motorcycles, reaching rates 2.0 times higher in the years that coincided with the pandemic (2019–2020).

Lastly, it was found that the total number of deaths at 24 h during the pandemic was similar to previous years, although the number of medical assistance had decreased significantly.

## Limitations

One of the main limitations of this study is that the exact make and engine capacity of the motorcycles involved in accidents were not analyzed. This data was not collected in the database of the Directorate of Traffic.

It would be interesting to know the accident rate based on the exact engine capacity. However, the database does not provide precise classification according to engine capacity; instead, we have included all motorcycles over 125 cubic centimeters.

Another limitation is the lack of access to data on patients admitted to Intensive Care Units (ICUs) who were victims of motorcycle accidents during the analyzed period. Individuals who passed away in the ICU within the first 24 h were not recorded in the DGT database, as they were not considered casualties and were excluded from the statistics. Having this information would have allowed us to obtain a more accurate understanding of the impact of accidents during the pandemic period and their association with the type of healthcare they might have received.

## Data Availability

The data used to support the findings of this study are available from the corresponding author upon request.
